# A Novel Tabletop Plane-Based Scapular Coordinate System Improves the Reproducibility of Preoperative Three-Dimensional Measurements in Reverse Total Shoulder Arthroplasty

**DOI:** 10.3390/jcm15208064

**Published:** 2026-10-17

**Authors:** Tomoya Manaka, Katsumasa Nakazawa, Yukihide Minoda, Rei Nishiura, Eriki Yanagi, Suguru Mori, Taisuke Nozu, Yoshihiro Hirakawa, Yoichi Ito, Hidetomi Terai

**Affiliations:** 1 Department of Orthopaedic Surgery, Graduate School of Medicine, Osaka Metropolitan University, Osaka 545-8585, Japan; manakatomoya@yahoo.co.jp (T.M.); reinishiura18@gmail.com (R.N.); miraculous.knabtfos@gmail.com (E.Y.); suguru.m.93@gmail.com (S.M.); nozu0504@icloud.com (T.N.); yoshimpreza@gmail.com (Y.H.);; 2 Department of Orthopaedic Surgery, Osaka Orthopedic Hospital, Osaka 553-0007, Japan; yminoda@omu.ac.jp; 3 Department of Orthopaedic Surgery, Ishikiriseiki Hospital, Higashiōsaka 579-8026, Japan; 4 Osaka Shoulder Center, Ito Clinic, Matsubara 580-0016, Japan; yito@omu.ac.jp

**Keywords:** reverse total shoulder arthroplasty, glenoid morphology, scapular coordinate system, three-dimensional measurement, reproducibility, intraclass correlation coefficient

## Abstract

**Background/Objectives:** Accurate three-dimensional (3D) assessment of glenoid morphology depends on the coordinate system used for measurement. Conventional landmark-based coordinate systems may be affected by observer-dependent variability in identifying the scapular landmarks. This study aimed to compare the reproducibility of a novel anterior scapular plane (ASP) coordinate system with that of the conventional surgical scapular plane (SSP). **Methods:** Twenty-five shoulders undergoing primary reverse total shoulder arthroplasty were evaluated using preoperative computed tomography scans. Glenoid version (GV), glenoid inclination (GI), glenoid height (GH), glenoid width (GW), and global offset (GO) were measured using both coordinate systems. Two fellowship-trained shoulder surgeons independently performed all measurements twice at a 1-month interval. Intra- and inter-observer reliability were assessed using intraclass correlation coefficients (ICCs). **Results:** ASP demonstrated almost perfect intra- and inter-observer reliability for all parameters, with ICCs ranging from 0.888 to 0.990 and from 0.864 to 0.932, respectively. In contrast, SSP demonstrated almost perfect intra-observer reliability (ICC range, 0.815–0.963) but only substantial inter-observer reliability for GV (ICC range, 0.660–0.705) and GI (ICC range, 0.660–0.738). Using ASP, inter-observer reliability improved to 0.887–0.924 for GV and 0.898–0.932 for GI. **Conclusions:** The ASP coordinate system demonstrated superior reproducibility compared with the conventional SSP coordinate system for preoperative 3D evaluation of glenoid morphology. Its anatomical accuracy and applicability to postoperative assessment remain to be validated.

## 1. Introduction

Reverse total shoulder arthroplasty (RTSA) is an effective treatment for glenohumeral arthritis, cuff tear arthropathy, and irreparable rotator cuff tears [1]. In recent years, the increasing use of RTSA has been accompanied by a rise in implant-related complications and revision surgeries [2,3,4]. Among these, the position of the glenoid component has a substantial influence on implant longevity, and superior inclination of the baseplate has been identified as a major cause of early failure [5,6]. Furthermore, recent studies have demonstrated considerable variability in glenoid morphology among patients, emphasizing the importance of accurately understanding scapular anatomy before surgery to achieve optimal glenoid component positioning [7].

Therefore, accurate assessment of glenoid morphology is essential for preoperative planning in shoulder arthroplasty. Axial two-dimensional (2D) computed tomography (CT) has become the standard imaging modality for preoperative evaluation of the glenoid because it provides greater accuracy and reproducibility than conventional radiography [8]. However, the accuracy of conventional 2D CT measurements of glenoid version (GV) is highly dependent on the relationship between the scapular plane and the CT scanner axis [9]. This relationship varies considerably with patient positioning and scapular orientation, and substantial differences in GV measurements have been observed according to scapular rotation and abduction [10].

Recent advances in three-dimensional (3D) CT planning software have enabled more accurate evaluation of glenoid morphology and implant positioning [11]. However, the scapular coordinate systems implemented in these software packages are not standardized [12], and considerable variability exists among them [13,14,15]. Much of this variability arises from the manual identification of anatomical landmarks required by commonly used systems, such as the conventional coordinate system originally described by Kwon et al. [16]. For example, the trigonum spinae has been reported to show only substantial inter-observer reproducibility (ICC = 0.76) because of its highly variable morphology, whereas other bony landmarks can be identified more consistently [17]. Because all 3D measurements depend on the coordinate system used, establishing a reproducible scapular coordinate system based on consistently identifiable landmarks is essential for accurate morphological assessment and preoperative planning.

To reduce observer-dependent variability, fully automated coordinate systems based on best-fit sphere algorithms and statistical shape modeling have recently been introduced and have demonstrated excellent reproducibility for preoperative planning [18,19]. However, because these automated systems rely on segmentation of the native scapular surface, the same coordinate system cannot be reliably reconstructed after shoulder arthroplasty, as implant placement, bone resection, and metal artifacts substantially alter the scapular morphology used for automated processing. Consequently, automated coordinate systems have limited utility for postoperative evaluation, precluding direct comparison between planned and achieved implant positions within a common anatomical reference frame. A coordinate system that provides high reproducibility and anatomical stability before and after arthroplasty is therefore needed.

To address this need, inspired by the anterior pelvic plane (APP), a highly reproducible anatomical reference used in total hip arthroplasty [20,21,22,23], we developed a novel scapular coordinate system based on three anterior anatomical landmarks: the anterior point of the superior angle, the anterior point of the inferior angle, and the anterior point of the coracoid process. These three points correspond to the points at which the scapula contacts a flat surface when placed with its anterior (costal) surface facing downward. The plane defined by these three points is therefore referred to as the tabletop plane. These landmarks were selected because they are expected to remain anatomically identifiable before and after shoulder arthroplasty.

The purpose of this study was to compare the intra- and inter-observer reliability of preoperative parameters obtained using the conventional coordinate system and the newly developed coordinate system. We hypothesized that the new coordinate system would demonstrate superior reproducibility and improved intra- and inter-observer reliability compared with the conventional method.

## 2. Materials and Methods

### 2.1. Ethics Approval

This study was approved by the Ethics Committee of Osaka City University Graduate School of Medicine (protocol code 3268; approved 1 December 2015) and by the Institutional Review Board of Osaka Metropolitan University Graduate School of Medicine (protocol code 2021-277; approved 28 March 2022).

### 2.2. Patients

Among the patients who underwent primary RTSA at our institution between December 2016 and September 2021, those whose preoperative CT covered the entire scapula and humerus with a slice thickness of 1 mm were included. Patients were excluded if they had a history of previous shoulder surgery, proximal humeral fracture or fracture sequelae, chronic locked dislocation, inflammatory arthritis such as rheumatoid arthritis, tumor, or infection; if they had severe glenoid bone loss corresponding to Walch types B2, B3, or C or Favard types E2 or E3; or if image quality was insufficient. Twenty-five patients (25 shoulders) met these criteria.

### 2.3. CT Acquisition and 3D Reconstruction

CT examinations were obtained at a single institution using an Aquilion ONE scanner (Canon Medical Systems, Otawara, Japan). CT images including the entire scapula and humerus were obtained with the following specifications: a tube voltage of 120 kV, a tube current determined by automatic exposure control, a helical pitch of 0.6, and a slice thickness of 1 mm, and were exported in Digital Imaging and Communications in Medicine (DICOM) format.

3D reconstruction and all measurements were performed using Zed Shoulder software (version 16.0.0; LEXI Co., Ltd., Tokyo, Japan). After manual digitization of the anatomical reference points, the software semiautomatically generated 3D models of the scapula and humerus by automatic segmentation. This software has also been used in previous computer simulation studies of RTSA [24,25].

### 2.4. Surgical Scapular Plane

**Figure 1:**
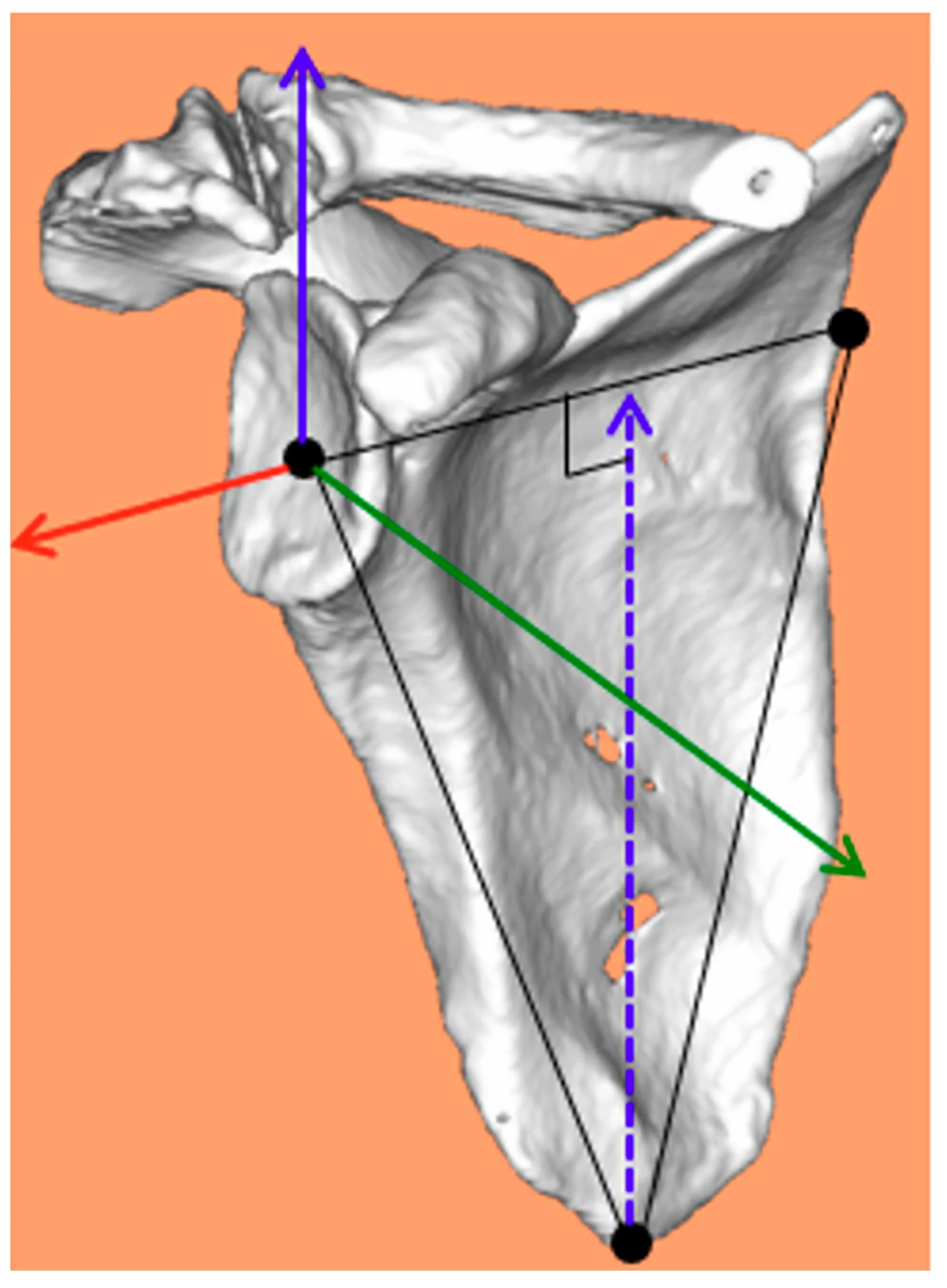
Coordinate system of the surgical scapular plane. The black triangle indicates the surgical scapular plane, and the red, green, and blue solid arrows indicate the X-, Y-, and Z-axes, respectively. The blue dashed arrow indicates the perpendicular line extending from the inferior angle of the scapula to the X-axis, which defines the direction of the Z-axis.

**Figure 2:**
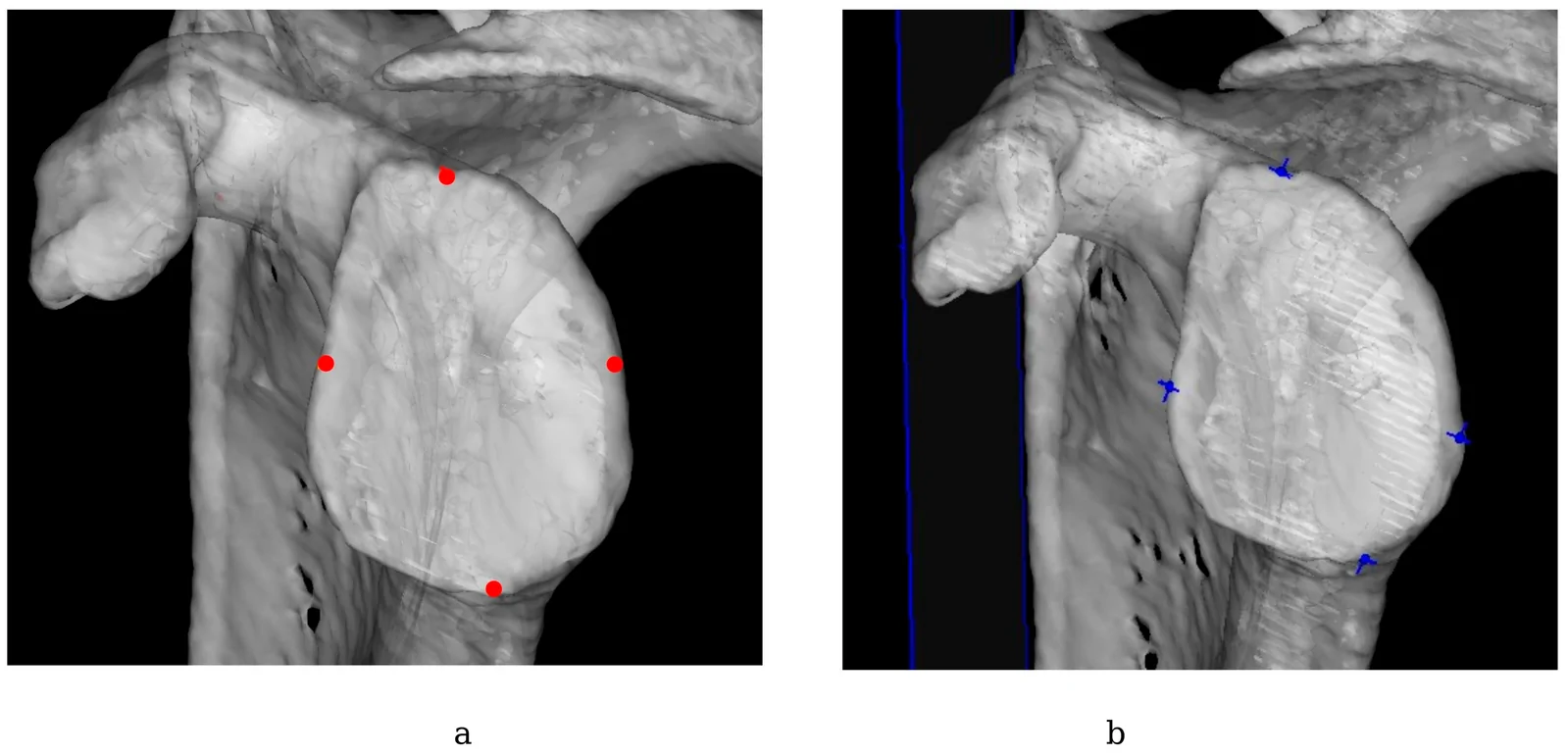
Comparison of manual and automated glenoid reference point determination in the surgical and anterior scapular planes. (**a**) Glenoid reference points in the surgical scapular plane; the red points indicate the reference points used for measurement. (**b**) Glenoid reference points in the anterior scapular plane; the blue points indicate the reference points used for measurement, and the blue straight line indicates the trace of the anterior scapular plane.

The conventional coordinate system was defined as the surgical scapular plane (SSP), based on the method originally described by Kwon et al. [16]. The origin was defined as the glenoid center. The X-axis was defined as the line connecting the glenoid center and the trigonum spinae—the most medial point of the scapular spine at its junction with the medial border of the scapula. The Z-axis was defined as the perpendicular line extending from the inferior angle of the scapula to the X-axis, and the Y-axis was defined as the axis perpendicular to the X- and Z-axes (Figure 1). The scapular plane was defined by the glenoid center, trigonum spinae, and inferior angle of the scapula. In the SSP coordinate system, the anterior, posterior, superior, and inferior glenoid reference points were manually identified by the observer (Figure 2a). These manually selected landmarks were subsequently used to calculate the glenoid morphological parameters.

### 2.5. Anterior Scapular Plane

**Figure 3:**
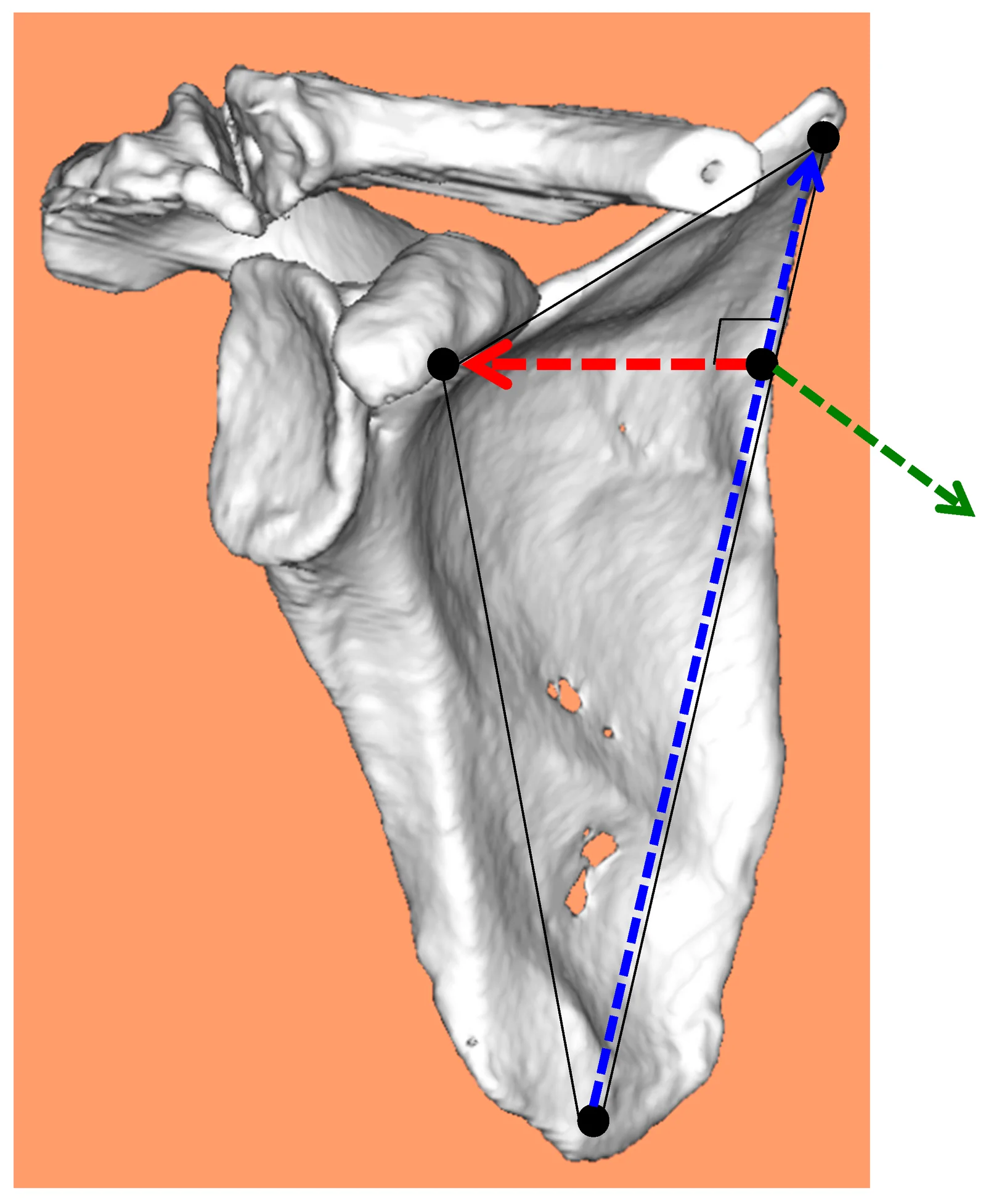
Coordinate system of the anterior scapular plane. The black triangle indicates the anterior scapular plane, and the red, green, and blue dashed arrows indicate the X-, Y-, and Z-axes, respectively.

**Figure 4:**
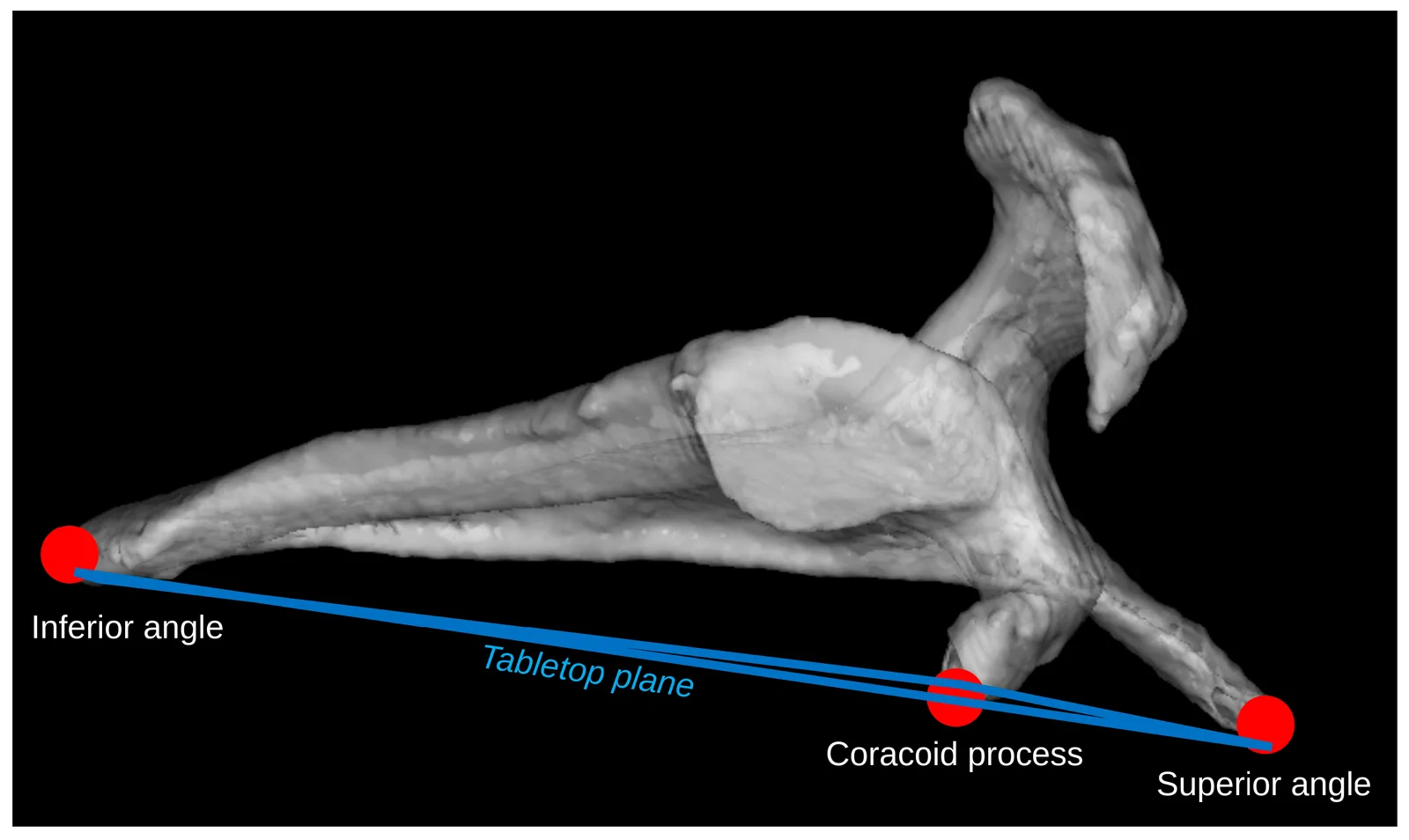
Definition of the tabletop plane. The three-dimensional scapular model is viewed approximately parallel to the anterior scapular plane. The three landmarks (red dots), representing the anterior points of the coracoid process, superior angle, and inferior angle, correspond to the points at which the scapula would contact a flat table when placed with its anterior (costal) surface facing downward. The blue lines indicate the trace of the tabletop plane. An anterior view of the same landmarks is shown in Figure 3.

The novel coordinate system was defined as the anterior scapular plane (ASP). The ASP was generated from a tabletop plane defined by three anterior anatomical landmarks: the anterior point of the superior angle of the scapula, the anterior point of the inferior angle of the scapula, and the anterior point of the coracoid process (Figure 3 and Figure 4). The tabletop plane represents the plane on which the scapula would rest if placed with its anterior (costal) surface facing downward on a flat table, with the three landmarks corresponding to the points of contact between the scapula and this plane (Figure 4). Each landmark was manually digitized by the observer on the 3D scapular model as the most anterior point of the respective structure. Although the morphology of the coracoid process varies among individuals, the landmark used for the ASP was defined not by a morphological feature of the coracoid process itself, such as its tip or base, but by the point at which it contacts the tabletop plane. Therefore, this landmark can be identified as a single point regardless of the overall shape of the coracoid process.

The line connecting the anterior points of the superior and inferior angles was defined as the Z-axis. The foot of the perpendicular line extending from the anterior point of the coracoid process to the Z-axis was defined as the origin. The line connecting the origin and the anterior point of the coracoid process was defined as the X-axis, and the Y-axis was defined as the axis perpendicular to the X- and Z-axes.

Using the ASP coordinate system, the anterior, posterior, superior, and inferior glenoid reference points were first digitized manually and then automatically adjusted by translating the coordinate planes along the ASP axes until they intersected the glenoid surface (Figure 2b). This adjustment was implemented in a research version of the software developed for the present study and is not a standard function of the commercially available system. Because this function was available only for the ASP, it was not applied to the SSP; the glenoid reference points in the SSP were selected manually according to the conventional method [16]. This process reduced the influence of manual landmark selection on the final position of the glenoid reference points.

### 2.6. Glenoid Morphological Measurements

The following parameters were measured using both coordinate systems:

- GV;
- Glenoid inclination (GI);
- Glenoid height (GH);
- Glenoid width (GW);
- Global offset (GO).

GV was defined as the angle between the scapular X-axis and the line connecting the midpoint of the anterior and posterior glenoid reference points to the center of the glenoid best-fit sphere, projected onto the XY plane of each coordinate system. Anteversion values were expressed as positive values and retroversion values as negative values.

GI was defined as the angle between the scapular X-axis and the line connecting the midpoint of the superior and inferior glenoid reference points to the center of the glenoid best-fit sphere, projected onto the XZ plane of each coordinate system. Superior inclination values were expressed as positive values and inferior inclination values as negative values.

The glenoid best-fit sphere was automatically calculated from the four glenoid reference points and the 3D scapular model using the software algorithm implemented in the Zed Shoulder planning system, based on the sphere-fitting method described by Lewis and Armstrong [18].

GH was defined as the distance between the superior and inferior glenoid reference points.

GW was defined as the distance between the anterior and posterior glenoid reference points. Because these points were not necessarily located at the same superoinferior level, GW represents the straight-line distance between them, which may be oblique, rather than the anteroposterior width measured perpendicular to the superoinferior axis of the glenoid.

For SSP, GO was defined as the difference in the X-coordinate between the glenoid center and the most lateral point of the greater tuberosity in the scapular coordinate system, calculated as the X-coordinate of the greater tuberosity minus that of the glenoid center.

For ASP, GO was defined as the perpendicular distance from the Z-axis to the most lateral point of the greater tuberosity and was automatically calculated by the software algorithm.

### 2.7. Intra-Observer and Inter-Observer Reliability

Preoperative CT images were independently evaluated by two fellowship-trained shoulder surgeons who were blinded to patient identity and each other’s measurements.

To assess intra-observer reliability, all measurements were repeated after a 1-month interval, with the observers remaining blinded to the initial measurements.

The order of the CT images was randomized for each assessment session to minimize recall bias.

### 2.8. Statistical Analysis

The mean values and standard deviations of all parameters were calculated from the first and second measurement sessions.

Normality was assessed using the Shapiro–Wilk test, and all parameters were normally distributed.

Intraclass correlation coefficients (ICCs) were calculated as described by Shrout and Fleiss [26]. ICC (1,1) was used to assess intra-observer reliability, and ICC (2,1) was used to assess inter-observer reliability.

ICC values were interpreted as follows [27]:

- Slight agreement: ≤0.20;
- Fair agreement: 0.21–0.40;
- Moderate agreement: 0.41–0.60;
- Substantial agreement: 0.61–0.80;
- Almost perfect agreement: 0.81–1.00.

Differences in GV, GI, GH, and GW between the ASP and SSP were analyzed using one-way repeated-measures analysis of variance (ANOVA). GO was excluded from the between-system comparison because it is defined differently in the two coordinate systems, and the absolute values are therefore not interchangeable.

A priori sample size calculation based on two raters, an expected ICC of 0.90, and a 95% confidence interval width of 0.20 demonstrated that a minimum of 14 shoulders would be required [28].

Statistical significance was set at *p* < 0.05. Statistical analyses were performed using SPSS version 25.0 (IBM Corp., Armonk, NY, USA).

## 3. Results

### 3.1. Patient Demographics

Nineteen shoulders were right shoulders, and six were left shoulders. There were 4 men and 21 women. The indications for surgery were massive irreparable rotator cuff tear without glenohumeral arthritis in 10 shoulders (Hamada grades 1–3) and cuff tear arthropathy in 15 shoulders (Hamada grades 4A and 4B).

The mean age at surgery was 78.8 ± 4.8 years (range, 69–89 years). The mean height was 150.3 ± 8.2 cm (range, 135.7–167 cm), the mean body weight was 56.7 ± 10.8 kg (range, 36.5–79 kg), and the mean body mass index (BMI) was 25.0 ± 3.6 kg/m^2^ (range, 17.3–30.8 kg/m^2^).

According to the Hamada classification [29], there were two Grade 1 shoulders, six Grade 2 shoulders, two Grade 3 shoulders, eight Grade 4A shoulders, and seven Grade 4B shoulders. According to the Walch classification [30], 23 shoulders were classified as type A1 and two as type A2. According to the Favard classification [31], 18 shoulders were classified as E0 and seven as E1.

### 3.2. Morphological Measurements

The results of all morphological measurements are summarized in Table 1.

Compared with SSP, measurements obtained using ASP showed significantly greater glenoid retroversion and GI. GH and GW were also significantly greater with ASP than those with SSP (all *p* < 0.05). GO was not statistically compared between the two coordinate systems because of the different definitions used; the mean GO was 43.2 ± 4.6 mm with SSP and 114.8 ± 10.7 mm with ASP.

### 3.3. Intra-Observer and Inter-Observer Reliability

The intra- and inter-observer reliability results for SSP and ASP are summarized in Table 2 and Table 3.

For SSP, intra-observer reliability for GV and GI was almost perfect for both observers, whereas inter-observer reliability was substantial in both measurement sessions.

Intra-observer reliability for GH and GW was almost perfect for both observers. Inter-observer reliability was almost perfect for GH and substantial for GW.

For GO, intra-observer reliability was almost perfect for both observers, whereas inter-observer reliability was substantial during the first measurement session and almost perfect during the second.

In contrast, ASP demonstrated almost perfect intra- and inter-observer reliability for all evaluated parameters.

For GV, inter-observer reliability improved from 0.660–0.705 with SSP to 0.887–0.924 with ASP. Similarly, inter-observer reliability for GI improved from 0.660–0.738 with SSP to 0.898–0.932 with ASP.

## 4. Discussion

In this study, the ASP coordinate system demonstrated superior intra- and inter-observer reliability compared with the conventional SSP coordinate system for preoperative 3D evaluation of glenoid morphology.

The number of RTSA procedures has increased substantially in recent years, making the reduction in complications essential for improving patient satisfaction and clinical outcomes. In particular, complications related to the glenoid component are among the most frequently reported causes of failure following RTSA. Preoperative planning is therefore critical for minimizing these complications, and previous studies have demonstrated that 3D planning and simulation of glenoid component placement can improve implant positioning and surgical accuracy [32].

Numerous preoperative planning software packages are currently used in shoulder arthroplasty. These systems employ different approaches to defining the scapular coordinate system, including landmark-based methods and automated algorithms based on statistical shape modeling [7,13,14,18,19]. Among landmark-based methods, reliability depends heavily on the anatomical landmarks used to construct the coordinate system. The trigonum spinae, in particular, has repeatedly been identified as the least reproducible landmark because of its variable morphology, with inter-observer ICCs as low as 0.76 [17]. Because 3D measurements are inherently dependent on the coordinate system used, differences in landmark reproducibility can translate directly into variability in measured glenoid morphology [13,14,15].

The conventional SSP coordinate system is generated using the glenoid center, inferior angle of the scapula, and trigonum spinae. This three-landmark method was originally described by Kwon et al., who validated it against cadaveric specimens and reported excellent inter- and intra-observer reliability (ICC = 0.983 and 0.978, respectively) for GV measurement. However, that validation involved only three experienced observers and 12 normal scapulae without structural deformity [16]. In the present study, intra-observer reliability for GV and GI was almost perfect, whereas inter-observer reliability was substantial. A separate study using a trigonum-based transverse axis for GI similarly reported an inter-observer ICC of only 0.87, compared with 0.96 for a transverse axis defined using five landmarks that did not rely on the trigonum [33]. The trigonum spinae used in SSP is often broad and ill-defined, potentially contributing to variability in landmark selection [34]. Furthermore, identification of the glenoid center may become difficult in patients with osteoarthritis because of glenoid deformity and erosion [35,36]. These factors may explain the lower intra- and inter-observer reliability observed with SSP.

As discussed in Section 1, fully automated systems provide excellent reproducibility for preoperative planning by estimating scapular orientation using best-fit sphere algorithms and statistical shape modeling without manual landmark identification [19]. However, because these methods rely on segmentation of the native scapular surface, the same coordinate system is difficult to reconstruct after shoulder arthroplasty, as implant placement, bone resection, and metal artifacts substantially alter scapular morphology. ASP was therefore designed as a landmark-based alternative that may be reconstructable in preoperative and postoperative settings because its three constituent landmarks lie outside the region typically altered by glenoid component implantation. A coordinate system reproducible across both settings would allow direct comparison of planned and achieved implant positions within a common anatomical reference frame, an approach previously used to quantify such discrepancies [37]. However, postoperative application of ASP was not evaluated in the present study, and further studies are warranted to confirm its postoperative reproducibility.

The ASP was developed as an anatomical landmark-based coordinate system inspired by the APP, which is widely used as a reproducible anatomical reference in total hip arthroplasty [20]. Jaramaz et al. demonstrated the utility of the APP for computer-assisted cup placement during hip arthroplasty [38], and APP has also been used to assess acetabular cup position postoperatively using CT, allowing direct comparison with the intraoperatively achieved position within the same anatomical reference frame [21]. Similarly, the ASP uses three consistently identifiable anterior scapular landmarks: the anterior point of the coracoid process, the anterior point of the superior angle, and the anterior point of the inferior angle. After manual identification of these anatomical landmarks, coordinate generation and determination of the glenoid reference points are performed automatically within the software environment. The use of landmarks that are relatively unaffected by glenoid deformity, together with the automated adjustment of the glenoid reference points, may have contributed to the excellent intra- and inter-observer reliability observed for the ASP in the present study; however, the relative contribution of each factor could not be determined.

In addition to reliability, the two coordinate systems produced significantly different mean values for GV, GI, GH, and GW, with these differences having direct clinical implications. Normative ranges and surgical planning targets established using SSP may not be directly transferable to ASP-based measurements. The differences in GV and GI are likely explained by the more anterior orientation of the ASP coordinate plane compared with SSP. Previous studies have similarly demonstrated that glenoid measurements vary according to the coordinate system used [13,14,15]. Because version and inclination are primary parameters used to guide glenoid component correction during surgical planning, clinicians should not apply SSP-derived thresholds, such as target retroversion or inclination angles, to ASP-based measurements without first establishing ASP-specific reference values.

The larger GH and GW values observed with ASP may be explained by differences in the methods used to determine the glenoid reference points. In the SSP, these landmarks were manually selected by the observer, whereas in the ASP, the manually digitized points were automatically adjusted by translating the coordinate planes along predefined axes (Figure 2b). These methodological differences may have contributed not only to differences in measurement values but also to the superior reproducibility observed with the ASP. It should be noted that GW in this study was defined as the distance between the anterior and posterior glenoid reference points, which may form an oblique line rather than represent the anteroposterior width measured perpendicular to the superoinferior axis of the glenoid. Therefore, GW as defined in this study does not necessarily correspond to the glenoid diameter used for baseplate size selection, and the GH and GW values reported in this study should be interpreted as parameters for assessing measurement reproducibility rather than as values to be applied directly to implant size selection.

The large difference in absolute GO values between ASP (114.8 ± 10.7 mm) and SSP (43.2 ± 4.6 mm) reflects a difference in definition rather than in landmark identification, and the two values were therefore not compared statistically. In SSP, GO was measured as the difference in the X-coordinate between the glenoid center and the greater tuberosity, whereas in ASP it was measured as the perpendicular distance from the Z-axis to the greater tuberosity. Because these definitions reference different anatomical origins, absolute GO values obtained with ASP and SSP are not directly interchangeable. GO should therefore be regarded as a coordinate-system-specific parameter rather than compared numerically between the two systems.

Some limitations should be acknowledged. First, the sample size was relatively small. However, as described in Section 2, our a priori sample size calculation indicated that at least 14 shoulders were required for the reliability analysis based on ICC calculations [28], and our study included 25 shoulders. Second, shoulders with severe glenoid bone defects were excluded; therefore, the cohort comprised only shoulders with minimal glenoid erosion (Walch types A1 and A2; Favard types E0 and E1). Although the three ASP landmarks are located away from the glenoid and are therefore not expected to be directly affected by glenoid erosion, severe deformity, such as biconcavity or marked posterior or superior erosion, may affect the automated adjustment of the glenoid reference points and the calculation of the glenoid best-fit sphere, potentially influencing the measured parameters [35,36]. Therefore, the present results cannot be extrapolated to shoulders with severe glenoid deformity, in which accurate preoperative planning is particularly critical, and further studies including such shoulders are warranted. Third, the glenoid reference points in the ASP were automatically adjusted by the software, whereas those in the SSP were manually selected, which may have introduced methodological bias favoring the ASP. Because this adjustment reduces observer-dependent variability in the final position of the glenoid reference points, some of the superior reproducibility of the ASP may be attributable to the higher degree of automation rather than to the coordinate system itself. The present study design did not allow these two effects to be separated. Future studies comparing both coordinate systems under identical conditions for glenoid reference point determination, either fully manual or fully automated, are required to isolate the contribution of the coordinate system. Finally, the glenoid reference points differed between ASP and SSP, which may have influenced the measurement values.

Most importantly, this study evaluated reproducibility rather than anatomical accuracy. Although ASP demonstrated superior intra- and inter-observer reliability, its relationship to the true anatomical scapular axis remains unknown. Further studies are warranted to validate the anatomical accuracy and clinical utility of this coordinate system and to evaluate its applicability to postoperative assessment, including the intra- and inter-observer reliability of postoperative measurements.

## 5. Conclusions

The ASP, a novel tabletop plane-based scapular coordinate system, demonstrated excellent intra- and inter-observer reliability for preoperative 3D scapular measurements. Compared with the conventional coordinate system, the ASP demonstrated superior reproducibility, particularly for GV and GI. Further studies are warranted to validate its anatomical accuracy and applicability to postoperative assessment.

## Figures and Tables

**Table 1:** Comparison of morphological parameters between the surgical and anterior scapular planes.

	Glenoid Version (°)	Glenoid Inclination (°)	Glenoid Height (mm)	Glenoid Width (mm)	Global Offset (mm)
Surgical scapular plane	−1.6 ± 3.8 (−14.2 to 5.9)	1.7 ± 5.5 (−9.9 to 15.8)	31.7 ± 3.5 (24.8–43.1)	24.0 ± 3.1 (17.5–31.8)	43.2 ± 4.6 (33.6–53.0)
Anterior scapular plane	−12.4 ± 6.0 (−30.2 to −0.9)	7.8 ± 5.9 (−7.6 to 22.7)	33.3 ± 5.1 (20.3–44.2)	27.4 ± 4.3 (16.3–35.9)	114.8 ± 10.7 (94.3–132.7)
*p* value	<0.01 *	<0.01 *	0.042 *	<0.01 *	N/A

Values are presented as mean ± standard deviation (range). * *p* < 0.05. Global offset is not statistically compared between the two coordinate systems because it is defined differently in each system (see Section 2.6). N/A, not applicable.

**Table 2:** Intra-observer and inter-observer reliability of measurements obtained using the surgical scapular plane.

	Glenoid Version	Glenoid Inclination	Glenoid Height	Glenoid Width	Global Offset
Intra-observer reliability					
Observer 1	0.815 (0.628–0.914)	0.892 (0.773–0.951)	0.895 (0.778–0.952)	0.914 (0.817–0.961)	0.933 (0.856–0.970)
Observer 2	0.860 (0.711–0.935)	0.819 (0.635–0.916)	0.963 (0.918–0.983)	0.910 (0.809–0.959)	0.855 (0.703–0.933)
Inter-observer reliability					
1st measurement	0.660 (0.361–0.835)	0.660 (0.373–0.833)	0.870 (0.703–0.943)	0.770 (0.515–0.895)	0.729 (0.466–0.872)
2nd measurement	0.705 (0.441–0.858)	0.738 (0.495–0.874)	0.871 (0.729–0.941)	0.764 (0.473–0.896)	0.839 (0.669–0.926)

Values are intraclass correlation coefficients (95% confidence intervals).

**Table 3:** Intra-observer and inter-observer reliability of measurements obtained using the anterior scapular plane.

	Glenoid Version	Glenoid Inclination	Glenoid Height	Glenoid Width	Global Offset
Intra-observer reliability					
Observer 1	0.901 (0.791–0.955)	0.970 (0.935–0.987)	0.977 (0.950–0.990)	0.956 (0.905–0.980)	0.990 (0.977–0.995)
Observer 2	0.888 (0.765–0.949)	0.952 (0.895–0.978)	0.958 (0.909–0.981)	0.951 (0.894–0.978)	0.938 (0.867–0.972)
Inter-observer reliability					
1st measurement	0.887 (0.761–0.949)	0.898 (0.783–0.954)	0.879 (0.745–0.945)	0.864 (0.687–0.940)	0.932 (0.853–0.969)
2nd measurement	0.924 (0.836–0.966)	0.932 (0.852–0.969)	0.878 (0.743–0.944)	0.914 (0.815–0.961)	0.900 (0.750–0.958)

Values are intraclass correlation coefficients (95% confidence intervals).

## Data Availability

The original contributions presented in this study are included in the article. Further inquiries can be directed to the corresponding author.

## Abbreviations

The following abbreviations are used in this manuscript:

RTSA	Reverse total shoulder arthroplasty
CT	Computed tomography
2D	Two-dimensional
3D	Three-dimensional
SSP	Surgical scapular plane
ASP	Anterior scapular plane
APP	Anterior pelvic plane
GV	Glenoid version
GI	Glenoid inclination
GH	Glenoid height
GW	Glenoid width
GO	Global offset
ICC	Intraclass correlation coefficient
DICOM	Digital Imaging and Communications in Medicine
ANOVA	Analysis of variance
BMI	Body mass index
